# Association between frontal fibrosing Alopecia and Rosacea: Results from clinical observational studies and gene expression profiles

**DOI:** 10.3389/fimmu.2022.985081

**Published:** 2022-08-24

**Authors:** Lin Liu, Yangmei Chen, Jiayi Chen, Yuzhou Xue, Tingqiao Chen, Yuxin Li, Xinyi Shao, Jin Chen

**Affiliations:** ^1^ Department of Dermatology, The First Affiliated Hospital of Chongqing Medical University, Chongqing, China; ^2^ Department of Cardiology and Institute of Vascular Medicine, Peking University Third Hospital, Beijing, China

**Keywords:** frontal fibrosing alopecia, rosacea, bioinformatics, differentially expressed genes, correlation analysis, immune infiltration

## Abstract

**Background:**

In recent years, frontal fibrosing alopecia (FFA), a type of scarring alopecia, has attracted increasing attention. Several studies have reported the frequent occurrence of rosacea in FFA; however, the association between FFA and rosacea and the underlying pathogenesis have not been thoroughly clarified. Thus, this study aimed to quantify these relationships and investigate their shared molecular mechanisms.

**Methods:**

We evaluated the association between FFA and rosacea by analyzing clinical data from nine observational studies. We then analyzed the gene expression profiles of FFA and rosacea. First, differential expression analysis and weighted gene co-expression network analysis were used to identify the common differentially expressed genes (DEGs). Later, we conducted a functional enrichment analysis and protein-protein interaction network and used seven algorithms to identify hub genes. Then, we performed a correlation analysis between the hub genes and the gene set variation analysis scores of common pathways in the gene set enrichment analysis (GSEA). The results were validated using different datasets. Finally, transcription factors were predicted and verified, and CIBERSORT and single-sample GSEA were used to estimate the infiltrating immune cells.

**Results:**

Patients with FFA had significantly higher odds for rosacea (pooled odds ratio [OR], 2.46; 95% confidence interval [CI], 1.78–3.40), and the pooled prevalence of rosacea in patients with FFA was 23% (95% CI, 14–23%). Furthermore, we identified 115 co-DEGs and 13 hub genes (*CCR5*, *CCL19*, *CD2*, *CD38*, *CD83*, *CXCL8*, *CXCL9*, *CXCL10*, *CXCL11*, *CXCR4*, *IRF1*, *IRF8*, and *PTPRC*). Seven pathways showed a high correlation with these hub genes. In addition, one TF, STAT1, was highly expressed in both diseases, and the results of the immune infiltration analysis indicated the importance of M1 macrophages and effector memory CD8+ T cells.

**Conclusion:**

This study contributes to the understanding of the relationship between FFA and rosacea, and based on the hub genes, we reveal the potential pathologies shared by the two diseases. This finding provides new insights of underlying molecular mechanisms and it may inspire future research on this comorbidity.

## Introduction

Frontal fibrosing alopecia (FFA) is a primary scarring alopecia of the frontotemporal zone and eyebrow and other facial hair loss (1). It places a burden on people’s appearance and psychology and might affect their quality of life. Some studies have reported the prevalence of FFA in some regions and some populations: the prevalence through data from New York City health care system is 0.015% (2), and that in Brazilian dermatologists is 4.9% (3), however, the global prevalence of FFA is still unknown. It has become increasingly prevalent during the last decade and is considered an emerging epidemic (4–6). The risk factors for FFA such as sunscreens and moisturizers are also controversial (7, 8).

As research on FFA has increased in recent years, and cutaneous comorbidities of FFA have been investigated. Of these, rosacea, a chronic inflammatory skin disease characterized by erythema, telangiectasia, papules, and pustules (9), has been reported in several FFA studies. However, the association between FFA and rosacea has not yet been fully elucidated.

In addition, in terms of pathogenesis, these two diseases remain to be elucidated. Hormones, autoimmunity, and genetic susceptibility are thought to play a role in FFA (10) and recent studies have suggested that it may involve an attack on the region of the hair follicle by immune-mediated inflammatory infiltrates, especially T helper type 1 (Th1)-biased CD8+ T lymphocytes (10, 11). The mechanism of rosacea has not been fully explained, and the current pathophysiological model involves Th1 and Th17 cell-mediated immune responses, excessive inflammation, neurogenic dysregulation, and vasodilation (9, 12). To date, inflammation and immune responses are known to be involved in the pathogenesis of FFA and rosacea (12–14), but the detailed mechanism of FFA complicated with rosacea remains unclear.

In this study, we aimed to clarify and quantify the relationship between FFA and rosacea through a comprehensive meta-analysis of observational studies. To shed light on the underlying common pathogenesis, we analyzed the common transcription feature and identified hub genes from the gene expression datasets of FFA and rosacea. This study was expected to provide new insights for the molecular mechanism of FFA complicated with rosacea.

## Materials and methods

### Literature search

A comprehensive literature search and meta-analysis were performed in accordance with the Preferred Reporting Items for Systematic Reviews and Meta-Analyses guidelines. We searched literatures published before February 27th 2022 on PubMed, Cochrane and Web of Science databases using the search term “frontal fibrosing alopecia” without other limitations.

### Study selection and eligibility criteria

Two authors (L.L. and Y.C.) independently examined all the searched results after removing duplicates through titles and abstracts. And additional articles were manually searched by checking reference lists of articles that included full-text review. Studies were included if they met the following criteria: (i) original observational studies and (ii) reporting the data on the association between FFA and rosacea: FFA prevalence in patients with rosacea, prevalence of rosacea in FFA, odds ratio (OR), or hazard ratio (HR). There were no restrictions on the language or region.

### Quality assessment and statistical analysis

We used the Newcastle–Ottawa scale to assess the quality of eligible studies. However, an adapted version was used in studies without a control group (15). A meta-analysis was conducted to obtain the pooled prevalence, ORs, or HRs. When the heterogeneity among the studies was identified as high (I^2^ > 50%), the random-effects model was chosen. Otherwise, a fixed-effect model was selected. If heterogeneity was high, subgroup analysis (if applicable) and sensitivity analysis were used to explore the source of heterogeneity. In addition, publication bias was assessed using Begg’s test. Significance was set at P < 0.05. All meta-analyses were conducted using STATA version 12.0 (StataCorp LP, College Station, Texas).

### Gene data source

The Gene Expression Omnibus (GEO) database was searched for the related gene expression datasets using the keywords “frontal fibrosing alopecia” or “rosacea”. The datasets to be included should be derived from human skin samples and be obtained from the same platform. Finally, GEO datasets numbered GSE65914 and GSE58934 were obtained. The GSE65914 dataset on the GPL570 consists of 38 rosacea skin samples and 20 healthy controls, and the GSE58934 dataset based on the GPL570 contains six skin samples, including three FFA lesion samples.

### Identification of differentially expressed genes

The limma R package was utilized to screen the DEGs from GSE65914 and GSE58934 with the following parameters: adj.P.Val < 0.05 and |log2FC| ≥ 0.5 (16). We then examined the intersection of DEGs in GSE65914 and GSE58934. The overlapping DEGs were regarded as DEGs upregulated or downregulated in both GSE65914 and GSE58934, rather than DEGs that were upregulated in one dataset but downregulated in another dataset. Venn diagrams were used to visualize the overlap of DEGs.

### DEGs in significant module of weighted gene co-expression network analysis

WGCNA was performed using the top 25% of genes with the largest variance in GSE65914 (17). First, to obtain a valid and reliable network, we tested whether there were outliers using hierarchical clustering analysis. Subsequently, the best power value was selected to convert the matrix of correlations to the adjacency matrix (topological overlap matrix [TOM]) by scale independence and mean connectivity. Then, the genes based on a TOM were clustered using the average-linkage hierarchical clustering method. Finally, similar modules were merged, and the correlation between modules and rosacea was calculated using Pearson’s correlation analysis to identify the significant modules. Moreover, visualized using the Venn diagram tool, we took the intersection of the overlapping DEGs with the genes in the most significant modules. We regarded the genes in the intersection as common DEGs, i.e., “co-DEGs.”

### Functional enrichment analysis, protein-protein interaction network, and hub genes

Based on co-DEGs, we performed Gene Ontology (GO) annotations to characterize biological properties, including molecular function, cellular component, and biological process (18). We also conducted Kyoto Encyclopedia of Genes and Genomes (KEGG) pathway enrichment analysis to determine functional attributes (19).

The PPI network was built using the STRING database and visualized using Cytoscape. Furthermore, we used the cytoHubba plug-in of Cytoscape to identify the hub genes (20). We selected seven algorithms, including MCC, MNC, Degree, EPC, EcCentricity, Closeness, and Radiality, to obtain the hub genes.

### Correlation between pathways and hub genes

We explored the relationship between the hub genes and pathways and attempted to quantify this correlation. The gene set enrichment analysis (GSEA) of the KEGG pathway gene set was conducted according to the expression of all genes in GSE65914 and GSE58934 separately (21). Then, we identified common pathways in the GSEA results for FFA and rosacea. Subsequently, we performed gene set variation analysis (GSVA) in GSE65914 and GSE58934 to obtain the GSVA scores of the common pathways in each skin sample (22).

Based on the expression levels of the hub genes and GSVA scores of the common pathways in each skin sample, we conducted a correlation analysis of the common pathways and hub genes and visualized them using a correlation heat map. The results were considered significant at P < 0.05.

### Validation of hub genes

To verify our hub genes, the expression levels of the identified hub genes were analyzed in GSE125733. The GSE125733 dataset on GPL11154 was obtained from seven FFA skin samples and seven matched healthy controls. The expression levels of the genes were compared using the t-test, and a P-value < 0.05 was considered significant. Subsequently, we conducted the GSVA in this dataset and conducted a correlation analysis between the hub genes and common pathways.

### Prediction and verification of TFs

The Transcriptional Regulatory Relationships Unraveled by Sentence-based Text Mining (TRRUST) database was used to predict the TFs of the hub genes and build transcriptional regulatory networks. Moreover, the expressions of TFs were verified by a t-test in GSE65914 and GSE125733. In addition, multiple-samples Virtual Inference of Protein-activity by Enriched Regulon analysis (msVIPER) was used to detected the activation of TFs in GSE65914 and GSE186075 (23). The GSE186075 dataset was on GPL21290, containing 36 FFA skin samples and 12 normal controls. And msVIPER could assess the protein activity through an enrichment statistical analysis based on gene expression data and the regulatory network provided by ARACNe (24).

### Infiltrating immune cells by CIBERSORT and single-sample GSEA

We used the R package “CIBERSORT,” a deconvolution algorithm based on gene expression profiles according to the known reference set LM22 (leukocyte signature matrix), to explore the immune infiltration in FFA and rosacea (25). The 22 immune cells included T cells, B cells, macrophages, dendritic cells, natural killer (NK) cells, monocytes, mast cells, eosinophils, and neutrophils. Furthermore, we collected the immune cell infiltration scores of each sample and analyzed the correlation between these scores and the expression levels of the hub genes.

**Figure 1:**
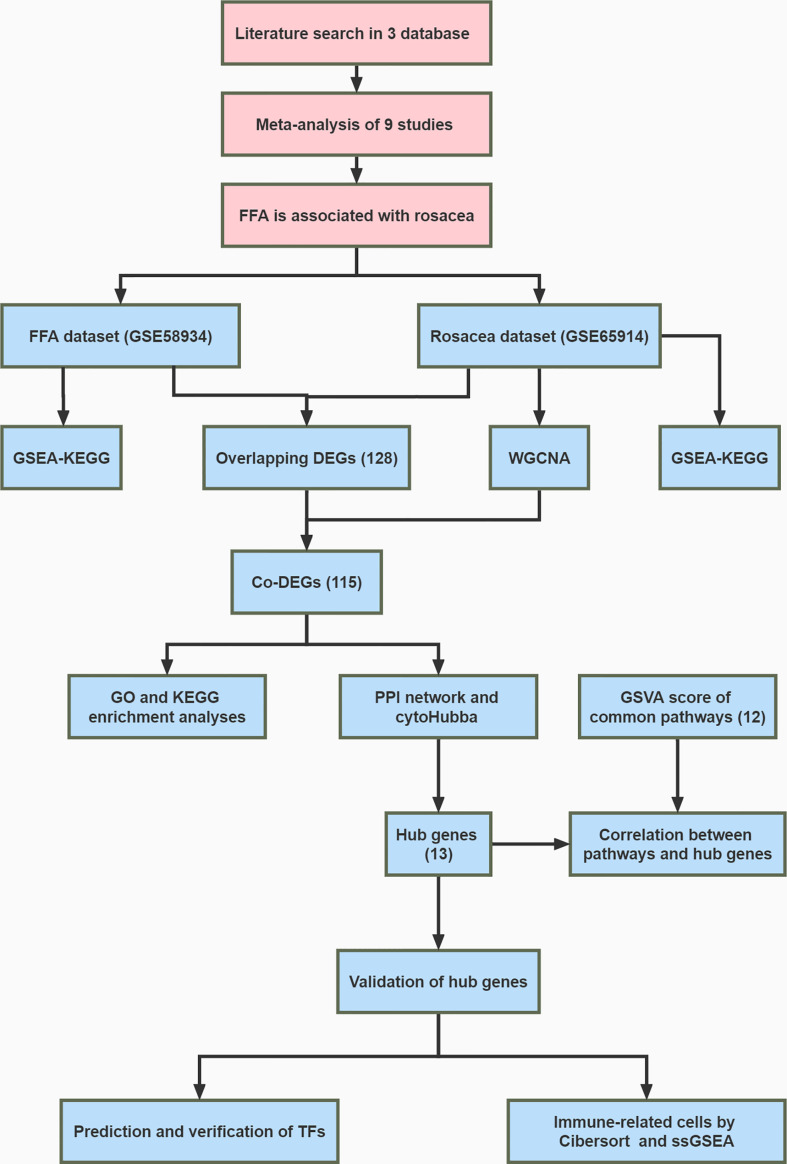
Flowchart of the whole study.

Then, ssGSEA was performed based on the expression levels of 29 immunity-associated signatures using the R package “GSEAbase.” We focused on the subtype of CD8 + T cells and examined the correlation between cells and hub genes. The whole research workflow is shown in **Figure 1**.

## Results

### Meta-analysis of observational studies

A total of 1379 articles were retrieved from PubMed, Cochrane, and Web of Science databases. After removing duplicates and screening the titles and abstracts, 119 studies were included in the full-text assessment stage. Finally, nine studies were eligible and included in the meta-analysis. The filtering process is illustrated in **Supplementary Figure 1**. Of these, six studies have reported cases of rosacea in patients with FFA, and the other studies have provided data on patients with FFA and controls. Therefore, we selected the prevalence and OR of rosacea among patients with FFA as the pooled subjects. More details regarding the characteristics of the eligible studies can be found in **Supplementary Table 1**.

**Figure 2:**
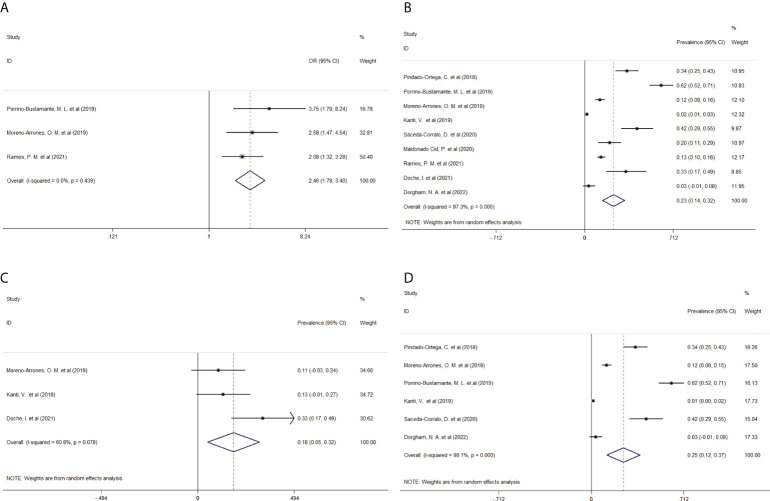
Forest plot of **(A)** the odds ratio of rosacea in patients with FFA, **(B)** pooled prevalence of rosacea in patients with FFA, **(C)** pooled prevalence of rosacea in male patients with FFA, and **(D)** pooled prevalence of rosacea in female patients with FFA. FFA, frontal fibrosing alopecia; OR, odds ratio; CI, confidence intervals.

Patients with FFA had higher odds of developing rosacea (pooled OR, 2.46; 95% CI, 1.78–3.40, I^2^ = 0) than those without FFA (**Figure 2A**). The pooled prevalence of rosacea in patients with FFA was 23% (95% CI, 14–23%, I^2^ = 97.3%) (**Figure 2B**). This result included 1674 patients with FFA from nine studies, and most (65%) of them were women aged >50 years. Subsequently, considering data availability, subgroup analyses of prevalence according to sex were performed. The pooled prevalence in male patients was 18% (95% CI, 5–32%, I^2^ = 60.8%), whereas that in female patients was 25% (95% CI, 12–37%, I^2^ = 98.1%) (**Figures 2C, D**). The sensitivity analysis confirmed the quality and stability of the results (**Supplementary Figure 2**). There was no publication bias in Begg’s test (**Supplementary Table 2**).

### Screening of co-DEGs

**Figure 3:**
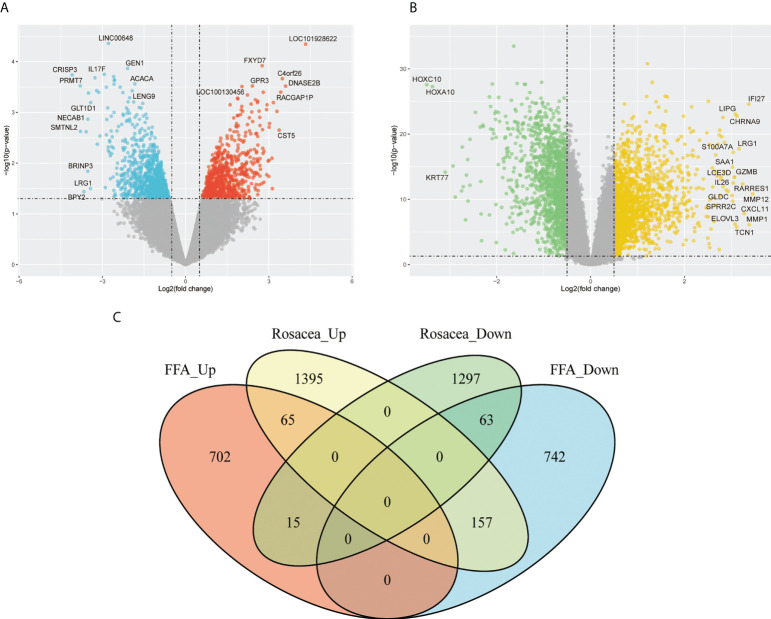
Identification of DEGs in FFA and rosacea: **(A)** volcano plot of the FFA dataset (GSE58934), **(B)** volcano plot of the rosacea dataset (GSE65914), and **(C)** Venn diagram of the overlap of DEGs between FFA and rosacea. DEGs, differentially expressed genes; FFA, frontal fibrosing alopecia.

Since we found that patients with FFA were prone to rosacea, we further explored their common mechanism. Based on the GSE65914 and GSE58934 datasets, 1744 DEGs related to FFA and 2992 DEGs related to rosacea were identified using the limma R package. As shown in the Venn diagram (**Figure 3**), 65 genes were upregulated and 63 genes were downregulated in both FFA and rosacea.

**Figure 4:**
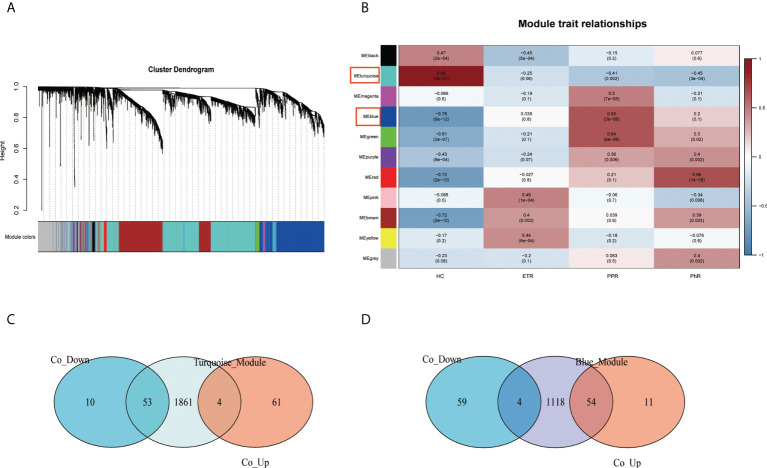
Construction of the weighted gene co-expression network analysis model for the GSE65914 dataset: **(A)** network heat map of all genes, **(B)** relationship between modules and trait, **(C)** Venn diagram of the turquoise module and the overlapping DEGs, and **(D)** Venn diagram of the blue module and the overlapping DEGs. DEGs, differentially expressed genes.

Through the construction of the WGCNA (**Figures 4A, B**), 11 modules were identified in GSE65914. The turquoise and blue modules were found to have the strongest relationship with rosacea. We took the intersection of the overlapping DEGs with genes in the turquoise module and the overlapping DEGs with genes in the blue module separately. We identified 115 co-DEGs by taking the intersection (**Figures 4C, D**).

### Functional enrichment analysis, PPI network, and hub genes

**Figure 5:**
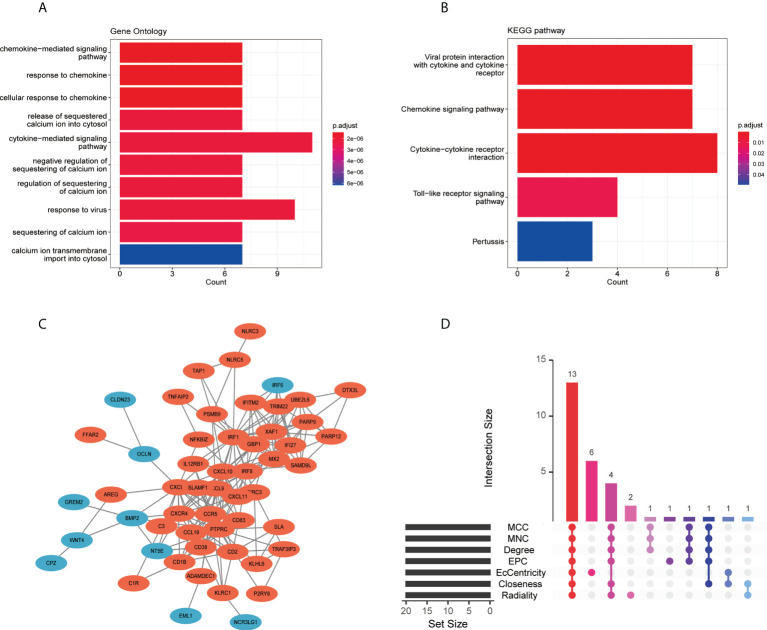
Functional enrichment analysis, PPI network, and screening of hub genes: **(A)** GO enrichment analysis of co-DEGs, **(B)** KEGG enrichment analysis of co-DEGs, **(C)** PPI network of co-DEGs, and **(D)** screening of hub genes showed by the upset diagram of seven algorithms. DEGs, differentially expressed genes; GO, gene ontology; KEGG, Kyoto Encyclopedia of Genes and Genomes; PPI, protein-protein interaction.

The GO analysis result for the 115 co-DEGs suggested that these genes were mostly enriched in the “cytokine-mediated signaling pathway,” “response to virus,” “calcium ion transmembrane import into the cytosol,” and “chemokine-mediated signaling pathway” (**Figure 5A**). As for the KEGG pathway, co-DEGs were enriched in the “cytokine-cytokine receptor interaction,” “viral protein interaction with cytokine and cytokine receptor,” “chemokine signaling pathway,” and “toll-like receptor signaling pathway” (**Figure 5B**).

The PPI network was built using the STRING database and Cytoscape (**Figure 5C**). Two gene clusters with scores >5 were focused on by MCODE based on the PPI network data (**Supplementary Figure 3**). Then, we used the cytoHubba in Cytoscape to determine hub genes, and the upset diagram of the seven algorithms showed 13 hub genes (**Figure 5D**), including *IRF1*, *CXCL8*, *CXCL9*, *CCR5*, *CXCR4*, *IRF8*, *CXCL10*, *PTPRC*, *CXCL11*, *CCL19*, *CD38*, *CD83*, and *CD2*. Details of the 13 hub genes are listed in **Supplementary Table 3**.

### Correlation between pathways and hub genes

**Figure 6:**
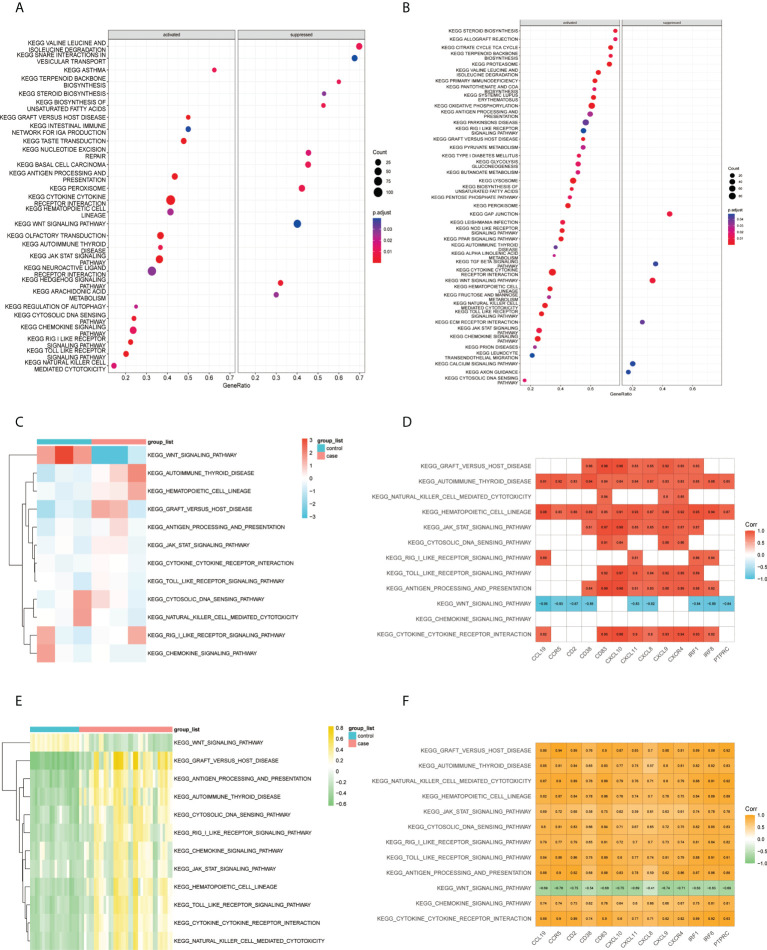
Correlation between pathways and hub genes: **(A)** GSEA–KEGG results of FFA, **(B)** GSEA–KEGG results of rosacea, **(C)** GSVA scores of the common GSEA pathways in FFA, **(D)** correlation heat map between the expression levels of hub genes and GSVA scores of the common pathways in FFA, **(E)** GSVA scores of FFA in common GSEA pathways, and **(F)** correlation heat map between the expression levels of hub genes and GSVA scores of the common pathways in rosacea. GSVA, gene set variation analysis; GSEA, gene set enrichment analysis; KEGG, Kyoto Encyclopedia of Genes and Genomes.

According to the results of the GSEA–KEGG analysis, there were 17 activated and 11 suppressed pathways in FFA and 37 activated and 6 suppressed pathways in rosacea (**Figures 6A, B**). We compared the two results and found 11 common activated pathways including the “toll-like receptor signaling pathway,” “JAK/STAT signaling pathway,” “autoimmune thyroid disease,” and “cytokine-cytokine receptor interaction.” One common suppressed pathway, the “Wnt signaling pathway,” was identified.

Furthermore, we studied the association between the common pathways and hub genes by correlating the GSVA scores (**Figures 6C, E**) of the pathways with the expression of hub genes in each sample. The results (**Figures 6D, F**) showed that in both FFA and rosacea, the expression level of hub genes was significantly positively correlated with the pathways including the “hematopoietic cell lineage,” “autoimmune thyroid disease,” “cytokine-cytokine receptor interaction,” “toll-like receptor signaling pathway,” “JAK/STAT signaling pathway,” “graft-versus-host disease,” and “antigen processing and presentation” and significantly negatively correlated with the “Wnt signaling pathway.”

### Validation of hub genes

**Figure 7:**
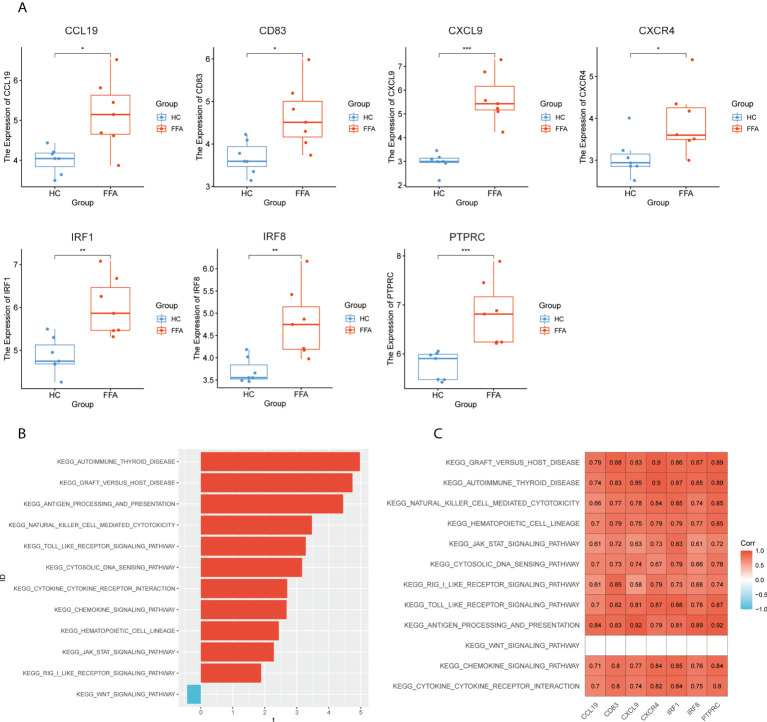
Validation of hub genes: **(A)** expression levels of hub genes in GSE125733, *p < 0.05, **p < 0.01, ***p < 0.001, ****p < 0.0001, **(B)** T-statistic of the GSVA scores in common GSEA pathways, and **(C)** correlation heat map between the expression levels of hub genes and GSVA scores of the common pathways in GSE125733. GSVA, gene set variation analysis; GSEA, gene set enrichment analysis.

To verify the hub genes, we analyzed the expression of hub genes in GSE125733 (**Figure 7A**). As shown in **Figure 7A**, *CCL19*, *CD83*, *CXCL9*, *CXCR4*, *IRF1*, *IRF8*, and *PTPRC* were significantly upregulated (P < 0.05). As the different platforms were used, the expressions of other hub genes were not detected.

Subsequently, the correlation analysis of hub genes and GSVA scores showed that hub genes had a significantly positive correlation with 11 common activated pathways including the “hematopoietic cell lineage,” “autoimmune thyroid disease,” “cytokine–cytokine receptor interaction,” “toll-like receptor signaling pathway,” “JAK/STAT signaling pathway,” “graft-versus-host disease,” and “antigen processing and presentation” in GSE125733 (**Figures 7B, C**). This finding is consistent with the above results.

### Prediction and verification of TFs

**Figure 8:**
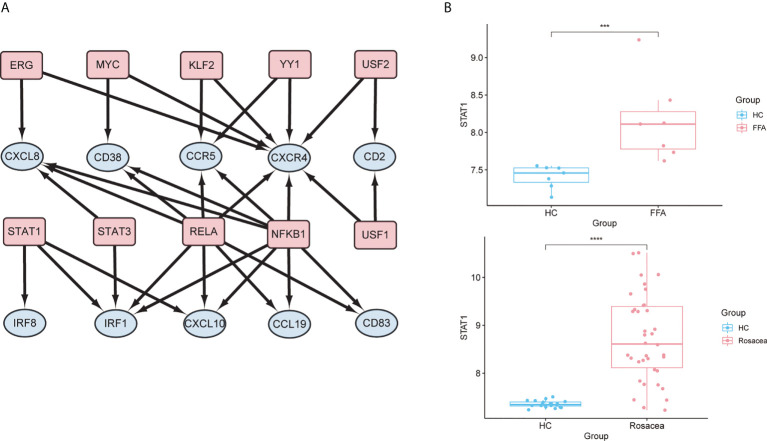
Prediction and verification of TFs: **(A)** TF regulatory network. TFs were marked in pink, and the hub genes were marked in blue. **(B)** The expression level of STAT1 in FFA and rosacea; ***p < 0.001, ****p < 0.0001. TF, transcription factor; FFA, frontal fibrosing alopecia.

In the TF regulatory network based on the TRRUST database, 10 TFs (RELA, STAT3, STAT1, ERG, YY1, KLF2, NFKB1, MYC, USF1, and USF2) were potentially associated with the regulation of hub genes (p < 0.05) (**Figure 8A** and **Supplementary Table 4**). Further verification of the GSE65914 and GSE125733 datasets was performed, and STAT1, which is involved in the regulation of three hub genes (*IRF1*, *IRF8*, and *CXCL10*), was found to be differentially expressed in FFA and rosacea (**Figure 8B**). And the result of msVIPER analysis in datasets GSE65914 and GSE186075 suggested that STAT1 was activated in both FFA and rosacea (P<0.05) (**Supplementary Figure 4**).

### Infiltrating immune cells by CIBERSORT and ssGSEA

**Figure 9:**
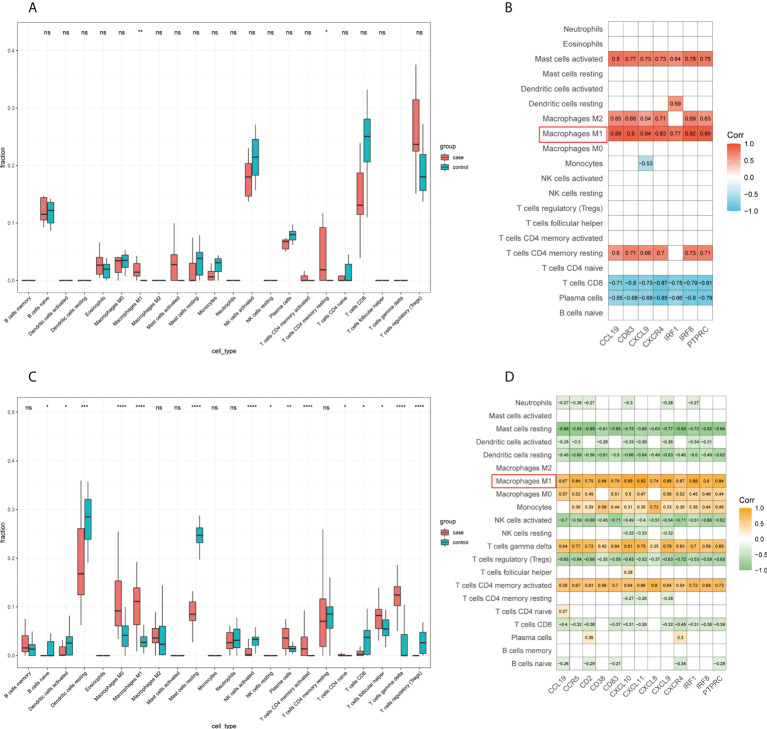
Infiltrating immune cells by CIBERSORT and its correlation with hub genes: **(A)** fraction of infiltrated immune cells in GSE125733, *p < 0.05, **p < 0.01, *** p < 0.001, **** p < 0.0001; **(B)** correlation heat map between the expression levels of hub genes and immune infiltration scores in GSE125733; **(C)** fraction of infiltrated immune cells in GSE65914, *p < 0.05, **p < 0.01, ***p < 0.001, ****p < 0.0001; and **(D)** correlation heat map between the expression levels of hub genes and immune infiltration scores in GSE65914. ns, no significance.

First, we detected the infiltration of immune cells in the GSE65914 and GSE125733 datasets using CIBERSORT. By comparing the CIBERSORT results in FFA and rosacea (**Figures 9A, C**), we found that M1 macrophages were significantly different between the rosacea skin samples and healthy controls and between FFA skin samples and healthy controls. Furthermore, we explored the relationship between the immune cells and hub genes. The CIBERSORT scores were used for the correlation analysis of the expression levels of the hub genes. Finally, the results for FFA and rosacea (**Figures 9B, D**) showed that M1 macrophages significantly positively correlated with all the hub genes (p < 0.05).

**Figure 10:**
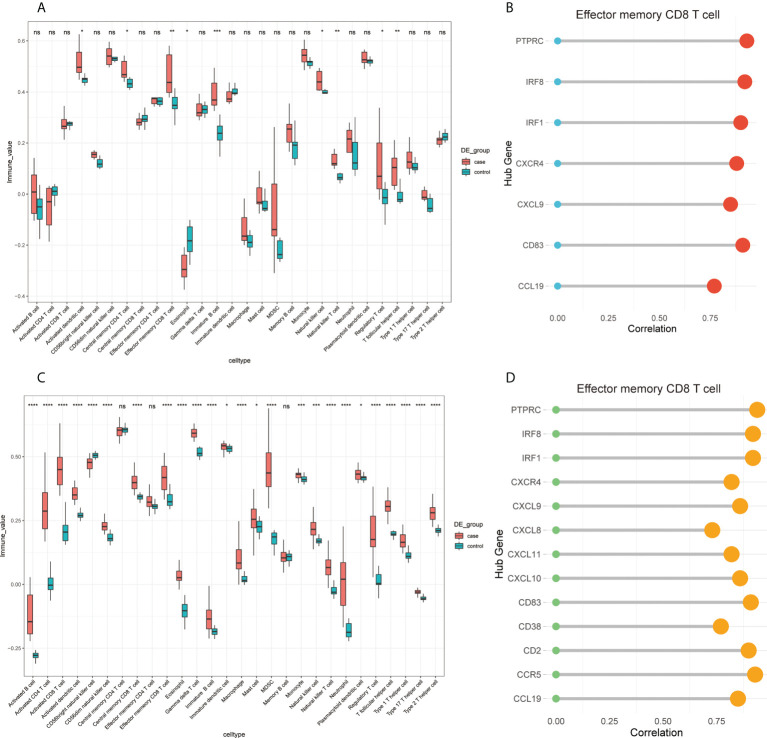
Infiltrating immune cells by ssGSEA and the correlation of effector memory CD8+ T cell and hub genes: **(A)** fraction of infiltrated immune cells in GSE125733, *p < 0.05, **p < 0.01, ***p < 0.001, ****p < 0.0001; **(B)** correlation between the expression levels of hub genes and immune infiltration scores of effector memory CD8+ T cells in GSE125733, *p < 0.05, **p < 0.01, ***p < 0.001, ****p < 0.0001; **(C)** fraction of infiltrated immune cells in GSE65914; and **(D)** correlation between the expression levels of hub genes and immune infiltration scores of effector memory CD8+ T cells in GSE65914. ns, no significance.

Considering that CIBERSORT analysis did not study the subtype of CD8+ T cells, we performed ssGSEA to study the immune infiltration of CD8+ T cells. The results of the ssGSEA in FFA and rosacea (**Figures 10A, C**) showed significant differences in activated dendritic cells, effector memory CD8+ T cells, eosinophils, immature B cells, NK cells, NK T cells, regulatory T cells, and T follicular helper cells between skin lesions and healthy controls. Moreover, we focused on effector memory CD8+ T cells and found that they were significantly related to hub genes through correlation analysis (**Figures 10B, D**).

Since both M1 macrophages and effector memory CD8+ T cells were found to be related to hub gene, we processed the correlation analysis between ssGSEA score of effector memory CD8+ T cells and CIBERSORT score of M1 macrophages by using a scatter plot. The coefficient of correlation in FFA samples was 0.84 (P<0.05), while the control group showed no significant correlation (**Supplementary Figures 5A, B**). And the result in rosacea samples was 0.74 (P<0.05), but there was still no significant relation in healthy controls (**Supplementary Figures 5C, D**).

In addition, we explored the correlation between common pathways and immune infiltration using CIBERSORT (**Supplementary Figure 6**). M1 macrophages were found to be associated with “graft-versus-host disease,” “JAK/STAT signaling pathway,” “toll-like receptor signaling pathway,” “antigen processing and presentation,” “cytokine-cytokine receptor interaction,” “hematopoietic cell lineage,” and “autoimmune thyroid disease” in both FFA and rosacea.

## Discussion

In this study, FFA was associated with rosacea (pooled OR, 2.46; 95% CI, 1.78–3.40) from clinical observational studies. Our meta-analysis suggested that the pooled prevalence of rosacea in patients with FFA was 23%, whereas the prevalence of rosacea in the general population was only 2.39% in a previous study (15). Individuals with FFA appear to be prone to rosacea, and the two diseases share some common clinical features, such as being more prevalent in adult women than in adult men, both of which are related to hormonal factors and smoking being a protective factor (15, 26–30). These results suggest that there may be some similarities and connections between the two diseases at the molecular level.

To explore the potential comorbidity mechanism of the two skin diseases, we further identified co-DEGs and found common TFs in the FFA and rosacea gene expression datasets. Finally, we identified 115 co-DGEs in both, 13 of which were hub genes, namely, *CCR5*, *CCL19*, *CD2*, *CD38*, *CD83*, *CXCL8*, *CXCL9*, *CXCL10*, *CXCL11*, *CXCR4*, *IRF1*, *IRF8*, and *PTPRC*. This shows that the development of these two diseases involves chemokines and cytokines, and these genes were significantly enriched in immune and inflammatory pathways. The GSEA and GSVA results suggested that in FFA and rosacea, seven pathways were significantly associated with hub genes, including the “toll-like receptor signaling pathway,” “JAK/STAT signaling pathway,” and “cytokine-cytokine receptor interaction.” Moreover, STAT1, which regulates three hub genes (*IRF1*, *IRF8*, and *CXCL10*), was highly expressed in these two diseases. In addition, M1 macrophages and effector memory CD8+ T cells were identified to be related to hub genes, and immune cell infiltration might play a role in the pathogenesis.

CCR5, CCL19, CXCL8, CXCL9, CXCL10, CXCL11, and CXCR4 are members of the chemokine superfamily, which consists of numerous ligands and receptors. Chemokines and their receptors play important roles in controlling leukocyte recruitment during inflammatory responses (31). Induced by interferon (IFN)-γ, CXCL9, CXCL10, and CXCL11 are usually secreted by monocytes, endothelial cells, fibroblasts, and keratinocytes (32, 33). These chemokines are all selective ligands for CXCR3 and lead to Th1 polarization. Previous studies have demonstrated strong Th1 skewing in FFA with significant upregulation of related markers, including IFNγ, CXCL9, and CXCL10 (34, 35). In rosacea, significantly higher expression levels of the Th1-signature cytokines, IFNγ, and tumor necrosis factor-α, also showed Th1 polarization (36). The IFNγ-related chemokines CXCL9, CXCL10, and CXCL11 had the highest expression levels among all chemokines in a study on rosacea by Buhl et al. (36). In addition, Liu et al. found that the serum concentrations of CXCL9 and CXCL10 were higher in patients with rosacea than in healthy controls, and CXCR3 in the inflammatory cells implicated in rosacea was elevated in Th1 cells (37). Th1 polarization related to the same chemokines might provide some clues for the pathogenesis of the coexistence of FFA and rosacea. In addition, FFA is described as an inflammatory condition that is only limited to the scalp after analysis of the proteomic FFA blood profile (34). In patients with rosacea, an increase in circulating serum chemokines CXCL9 and CXCL10 was found, which might provide evidence that rosacea is systematically involved. Essentially, many comorbidities of rosacea, such as Alzheimer’s disease (38) and inflammatory bowel disease (39) have shown an association with chemokines CXCL9 and CXCL10 in the circulating serum. Therefore, it is worth investigating whether there is an increase in these chemokines in the serum of patients of FFA complicated with rosacea.

Th1 cells can produce IFN-γ to promote immune cell migration, including cytotoxic lymphocytes, NK cells, NK T cells, and macrophages (33). Histopathologically, the number of CD8+ T cells in the follicles of patients with FFA increased (35, 40). On the contrary, the density of cytotoxic CD8+ T cells in rosacea is sparse (41, 42). Although it seems contradictory, ignoring the subtype of CD8+ T cells might be the explanation of it. Our results for immune cell infiltration emphasized the role of effector memory CD8+ T cells in FFA and rosacea. Memory CD8+ T cells could be present in skin and are able to provide rapid protection (43). And in other skin diseases, effector memory CD8+ T cells have been found to be more cytolytic and have the capacity to express chemokine receptors and rapidly produce large amounts of IFN-γ (44–46). The similar situation might occur in FFA and rosacea, but the problem on the subtype of CD8+ T cells in the two diseases still requires further exploration. As for macrophages, Harries et al. found macrophage polarization might play an important role in FFA (47). And hematoxylin and eosin staining and immunohistochemical staining of skin samples of rosacea showed higher expression of CD86 and profound infiltration of macrophages in previous study (48). High expression of CD86 indicates activated macrophage M1 in skin, which is mainly involved in proinflammatory responses (49). This is consistent with our results on immune cell infiltration. Compared with M2 macrophages, M1 macrophages could be activated by inflammatory cytokines such as IFN-γ and expressed chemokines and TNF-α (50). It is possible that M1 macrophages and effector memory CD8+ T cells has a crosstalk *via* the chemokines and cytokines. In fact, in previous studies, M1 macrophages have been found to produce chemokines to influence effector memory CD8+ T cells (51, 52). And increased levels of various cytokines could contribute to the generation of effector memory CD8+ T cells (53). We suppose that similar crosstalk of M1 macrophages and effector memory CD8+ T cells might happen in FFA and rosacea. And our result of the correlation analysis between ssGSEA score of effector memory CD8+ T cells and CIBERSORT score of M1 macrophages suggested that FFA and rosacea strengthened the correlation between the immune infiltration scores of these two types of cells, which might provide some clues to the crosstalk between them.

In addition, we identified the critical role of IRF1 and IRF8 in FFA and rosacea and the high expression of STAT1 in this study. And further msVIPER analysis confirmed the activation of STAT1 in FFA and rosacea. IRF8 and IRF1 are transcriptional regulators that activate macrophages *via* proinflammatory signals such as IFN-γ (54). STAT1, a member of the STAT family, is activated by Janus kinase upon IFN-γ stimulation (55). IRF1, IRF8, and STAT1 are important in human inflammatory diseases including systemic sclerosis, systemic lupus erythematosus, and inflammatory bowel disease (56, 57). In previous studies on FFA, STAT1 and IRF1 were found to be elevated in FFA lesioned skin compared with normal skin (34, 35), but few articles explored phosphorylation of STAT1 in FFA. The activated STAT1 can lead to upregulation of proinflammatory chemokines in epidermis (58), including CXCL9 and CXCL10 (59). In our studies, the result of msVIPER analysis supported the activation of STAT1 and many chemokines were found to be increased in FFA lesions. Besides, STAT1 and phosphor-STAT1 are primarily observed in the epidermis of inflammatory skin diseases, including lichen planus, psoriasis vulgaris, cutaneous lupus erythematosus and Hidradenitis suppurativa (60–62). Of them, lichen planus, which is considered to have histological similarities with FFA (63), has the same keratinocyte-derived STAT1 expression pattern and it plays a role in the pathogenesis of lichen planus (60). Furthermore, Blazanin N et al.’s study found that in mice with keratinocyte‐specific STAT1 deletion, induction of IRF‐1 and expression of proinflammatory chemokines were all markedly diminished (59). These results provide some clues that keratinocyte-derived STAT1 and its phosphorylation might play a role in the pathogenesis of FFA. As for rosacea, Deng Z et al. found the increased nuclear localization of phosphor-STAT1 in epidermal cells through immunohistochemistry of rosacea lesions (64). And they confirmed that STAT1 was the potential core TF of keratinocyte-immune cell crosstalk through epidermal RNA-Seq data. There is an overactive keratinocyte–macrophage crosstalk *via* the epidermal-derived IFN-γ/STAT1/IRF1 signature in rosacea, which might be an argument for the activation of the innate immune system for rosacea development (64). And the IFN-γ/STAT1/IRF1 pathway is related to macrophage polarization and STAT1 plays a key role in M1 polarization (65–67). Therefore, the IFN-γ/STAT1/IRF1 pathway might regulate M1 polarization in the presence of FFA and rosacea. IRF8 may play a supporting role in this process, such as the maintenance of steady-state epigenetic and transcriptional levels of critical macrophage pathways (54).

Since burning and pruritus are usually present on the scalp of patients with FFA and on the face in those with rosacea, there may be some similarities between the two diseases in terms of neurogenic inflammation. One explanation for the pathogenesis of FFA and rosacea may be neurogenic inflammation as reported in previous studies (68, 69). One clue is that neuropeptides, such as substance P and calcitonin gene-related peptide (CGRP), are abnormally expressed in both diseases, especially CGRP (13, 70). Another clue is that the number of mast cells, along with the proportion of degranulating cells, is increased in the perifollicular bulge region in FFA and the skin in rosacea (10, 70, 71). In addition, CXCR4 is involved in the migration of mast cells (72) and in this study, the expression of CXCR4 was significantly increased in FFA and rosacea. In skin neurogenic inflammation, CGRP induces mast cells to release vasoactive amines (73). In studies on other tissues and cells, CGRP was shown to play a role in the expression of CXCR4 (74, 75), although the details of this process are still unclear. Therefore, there might be a complicated interplay between CXCR4, mast cells, and neuropeptides in the neurogenic inflammation of FFA and rosacea. Notably, neurogenic inflammation related to mast cells and CGRP seems to be more associated with ETR than with other subtypes of rosacea (70). This may explain why ETR was the most frequent subtype of rosacea in patients with FFA in observational clinical studies (76, 77). Thus, the relationship between rosacea subtypes and FFA is worth exploring in the future.

However, our study had some limitations. Given the limited clinical data, there are no data available to evaluate FFA in patients with rosacea. In addition, our results lack further verification *in vitro*, and further external verification is needed. Thus, considering that our study is a preliminary exploration of the shared mechanism of FFA and rosacea, we hope to provide some meaningful directions for future research.

In summary, we found through a meta-analysis of clinical observational studies that patients with FFA are prone to rosacea. After analyzing the transcription datasets of FFA and rosacea, we identified 115 common DEGs and 13 hub genes. We found that the mechanisms of the two diseases had some overlap in inflammatory and immune responses. Further studies on the comorbidity of FFA and rosacea are needed. This study might provide new understanding into the biological mechanisms of these two diseases, and the hub genes identified in this study might serve as potential therapeutic targets in further studies.

## Data availability statement

The original contributions presented in the study are included in the article/**Supplementary Material**. Further inquiries can be directed to the corresponding author.

## Ethics statement

Ethical review and approval was not required for the study on human participants in accordance with the local legislation and institutional requirements. Written informed consent from the participants’ legal guardian/next of kin was not required to participate in this study in accordance with the national legislation and the institutional requirements.

## Author contributions

Conceptualization, LL and JC. Data acquisition and processing, YX and JYC. Interpretation of data, TC and YL. Software, XS and LL. Validation, LL, YC, and JYC. LL and YC were responsible for writing the initial article. Revision and finalization, LL and YC. All authors contributed to the article and approved the submitted version.

## Funding

This study was supported by National Natural Science Foundation of China (n82073462).

## Acknowledgments

We are very grateful to the authors of the GSE58934, GSE65914, GSE186075, and GSE125733 datasets; we thank the authors of the nine observational studies included in this work; we thank Weifeng Hong PhD, Department of Radiation Oncology, Zhongshan Hospital Affiliated to Fudan University, for his support throughout this work.

## Conflict of interest

The authors declare that the research was conducted in the absence of any commercial or financial relationships that could be construed as a potential conflict of interest.

## Publisher’s note

All claims expressed in this article are solely those of the authors and do not necessarily represent those of their affiliated organizations, or those of the publisher, the editors and the reviewers. Any product that may be evaluated in this article, or claim that may be made by its manufacturer, is not guaranteed or endorsed by the publisher.
