# Supplementary material for: Association between frontal fibrosing Alopecia and Rosacea: Results from clinical observational studies and gene expression profiles
Source: Front Immunol. 2022 Aug 24;13:985081. doi: 10.3389/fimmu.2022.985081 (PMC9448884; doi:10.3389/fimmu.2022.985081)
Supplement: Supplementary file 1 [file DataSheet_1.docx]

Supplementary Material

# Supplementary Tables

**Supplementary Table 1**. Characteristics of the included studies and Newcastle–Ottawa scale quality assessment.

**Supplementary Table 2**. Quantitative assessment results of Begg’s test.

**Supplementary Table 3**. Details of the hub genes.

**Supplementary Table 4**. Key transcription factors found on the transcriptional regulatory relationships unraveled by sentence-based text-mining database.

# Supplementary Figures


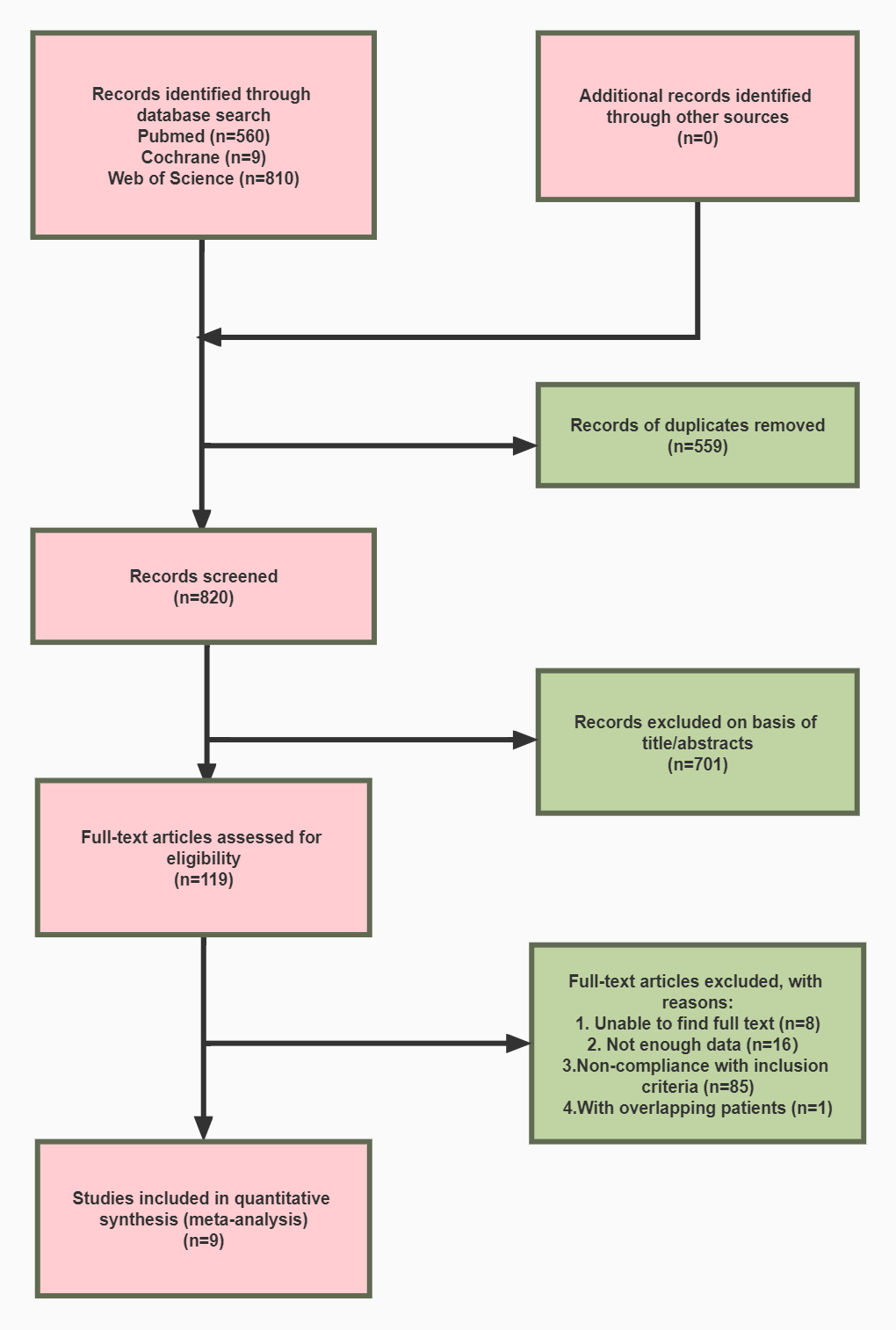


**Supplementary Figure 1**. Flow chart of the selection process for the studies included in the meta-analysis.


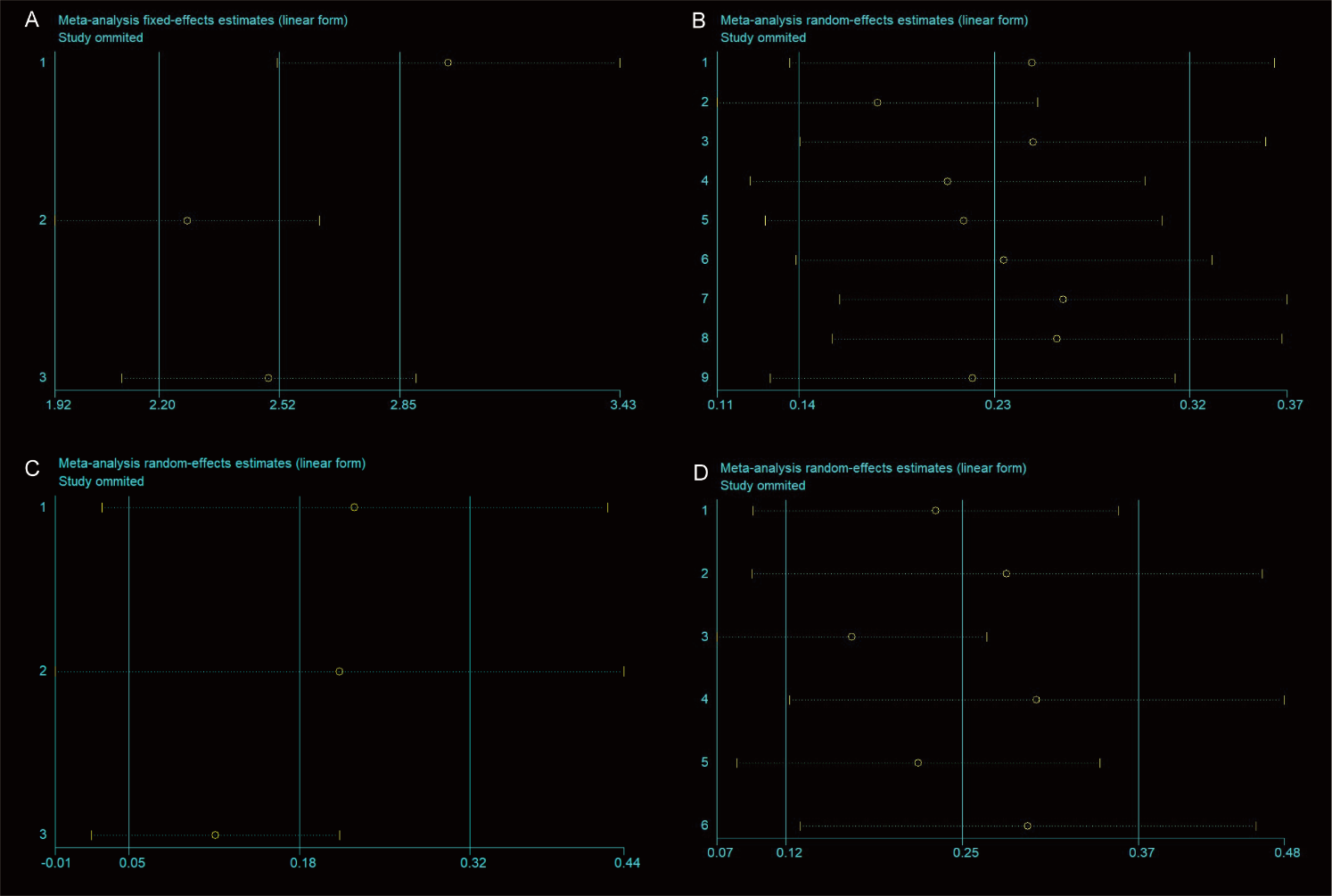


**Supplementary Figure 2**. Sensitivity analysis: (A) odds ratio of rosacea in patients with FFA, (B) pooled prevalence of rosacea in patients with FFA, (C) pooled prevalence of rosacea in male patients with FFA, and (D) pooled prevalence of rosacea in female patients with FFA. FFA, frontal fibrosing alopecia


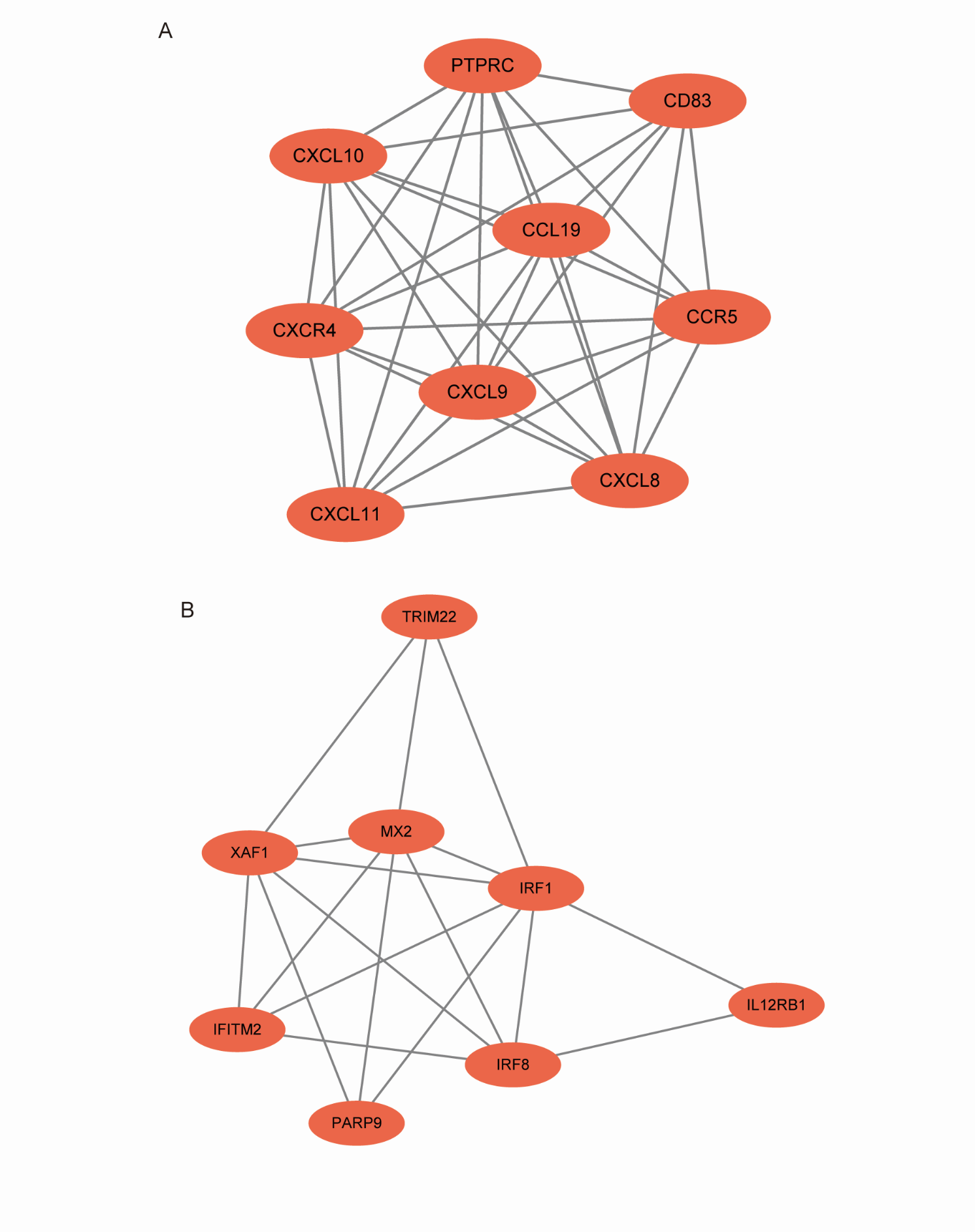


**Supplementary Figure 3**. Two significant gene clustering modules by MCODE based on the PPI network. (A) Cluster 1 (score = 8.75). (B) Cluster 2 (score = 5.143). PPI, protein-protein interaction


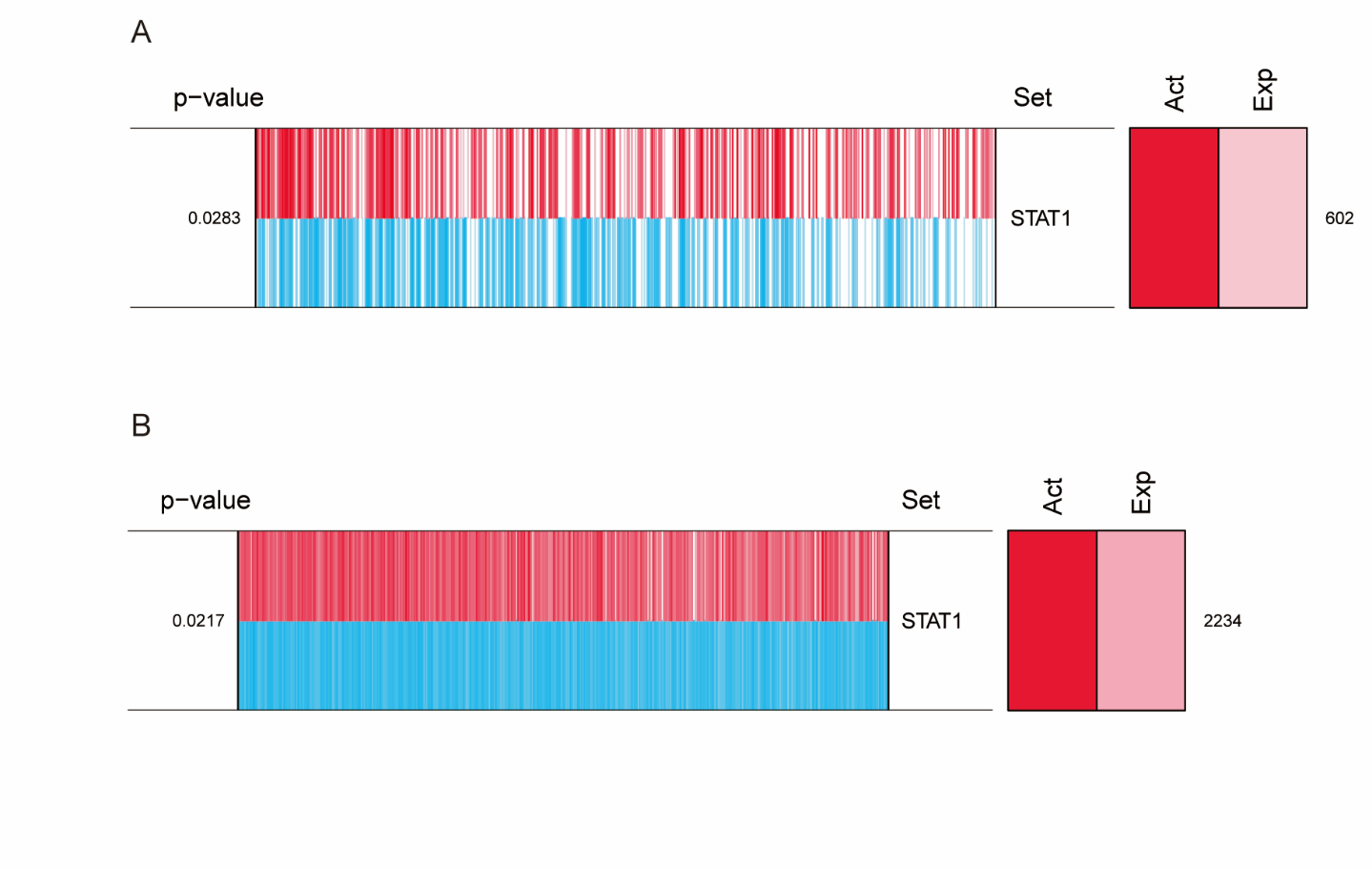


**Supplementary Figure 4.** MsViper analysis of STAT1 activation: (A) the STAT1 activation in FFA based on MsViper analysis in GSE186075, and (B) the STAT1 activation in rosacea based on MsViper analysis in GSE65914. MsViper, multiple-samples Virtual Inference of Protein-activity by Enriched Regulon analysis. FFA, frontal fibrosing alopecia


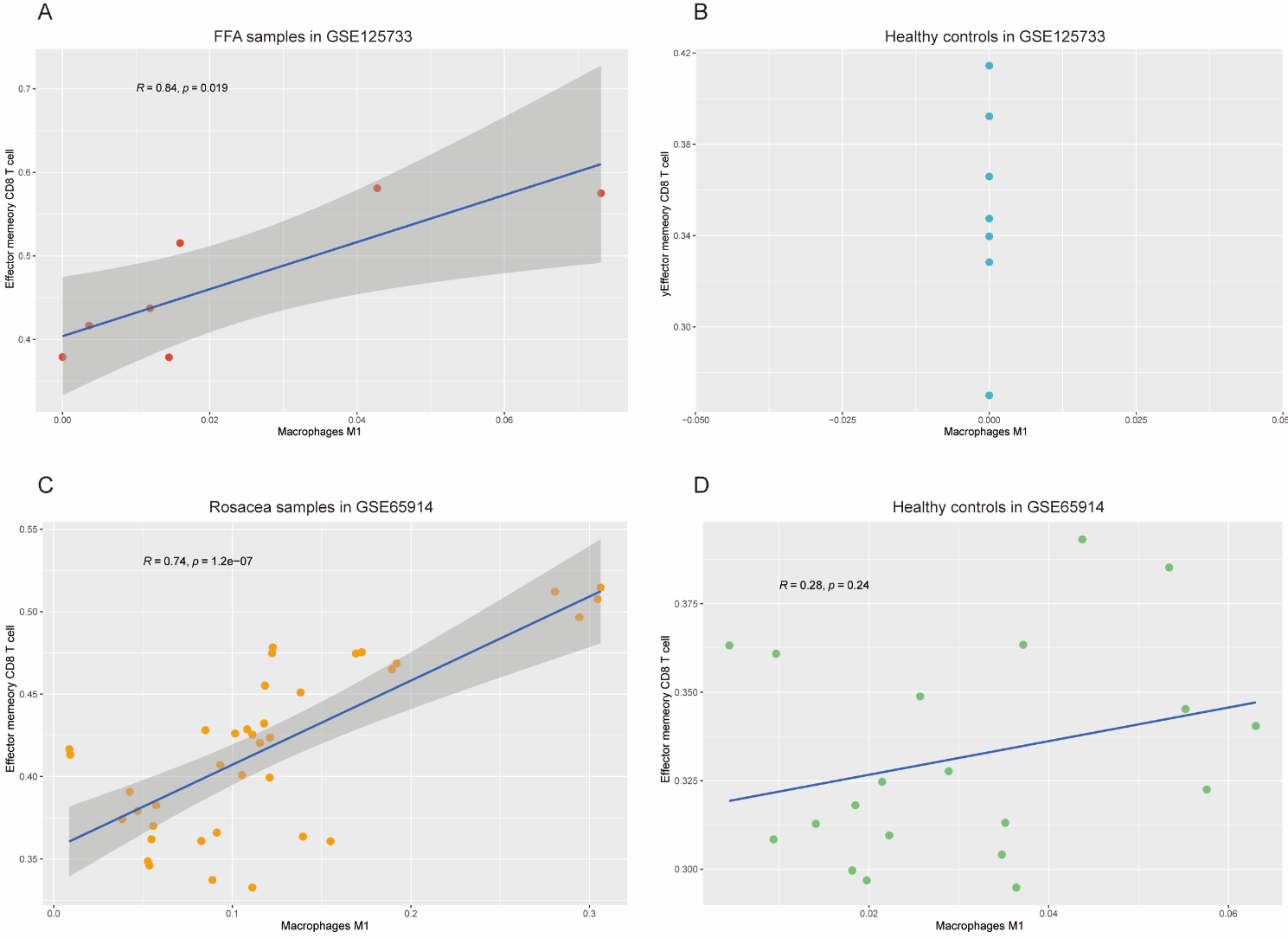


**Supplementary Figure 5.** The correlation analysis between ssGSEA score of effector memory CD8+ T cells and CIBERSORT score of M1 macrophages: (A) the scatter plot of FFA samples in GSE125733, (B) the scatter plot of healthy controls in GSE125733, (C) the scatter plot of rosacea samples in GSE65914, and (D) the scatter plot of healthy controls in GSE65914. FFA, frontal fibrosing alopecia


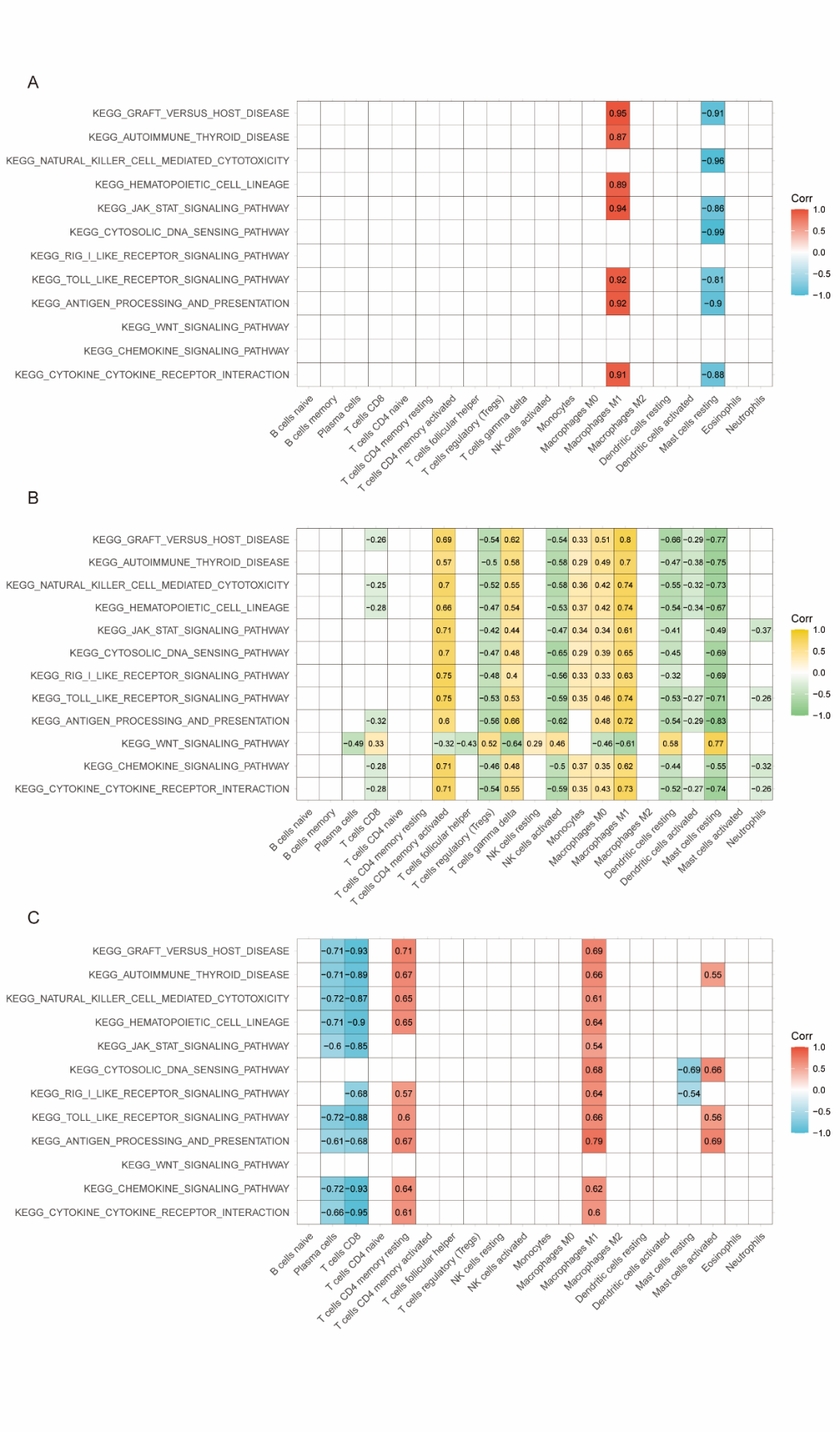


**Supplementary Figure 6.** Correlation between pathways and infiltrating immune cells by CIBERSORT: (A) correlation heat map between the GSVA scores and immune infiltration scores in GSE58934, (B) correlation heat map between the GSVA scores and immune infiltration scores in GSE65914, and (D) correlation heat map between the GSVA scores and immune infiltration scores in GSE125733. GSVA, gene set variation analysis
