# Supplementary material for: Association between frontal fibrosing Alopecia and Rosacea: Results from clinical observational studies and gene expression profiles
Source: Front Immunol. 2022 Aug 24;13:985081. doi: 10.3389/fimmu.2022.985081 (PMC9448884; doi:10.3389/fimmu.2022.985081)
Supplement: Supplementary file 2 [file Table_1.docx]

**Supplementary Table 1. Characteristics of the included studies and Newcastle–Ottawa scale quality assessment**

| Author | Year | Country | Study design | Assessment | FFA group | Control group | OR, 95% CI | Sex | Mean age/ age range | Source of FFA | Source of control | NOS score |
| --- | --- | --- | --- | --- | --- | --- | --- | --- | --- | --- | --- | --- |
|  |  |  |  |  | (event/total) | (event/total) |  | (M/F) |  |  |  |  |
| Ramos, P. M. et al | 2021 | Brazil | Case-control | By dermatologist | 60/451 | 31/451 | 2.079(1.319-3.277) | Sex matched: 17/434 | 53/49 | Prospectively recruited patients | Other patients | 7 |
| Porriño-Bustamante, M. L. et al | 2019 | Spain | Cross-sectional | By dermoscopic | 61/99 (ETR: 54; PPR: 7) | 12/40 | 3.746(1.703 -8.239) | All women | 63.3/61.7 | Clinic patients | Other patients | 8 |
| Moreno-Arrones, O. M. et al | 2019 | Spain | Case–control | By dermatologist | 38/308(M: 2/19; F: 36/289) | 20/387(Male: 0/58; Female: 20/289) | 2.583(1.470-4.538) | FFA: 19/289 Control: 58/289 | Male: 52.3/49.7; Female: 60/58.4 | Prospectively recruited patients | Other patients, close contacts and unrelated people | 9 |
| Saceda-Corralo, D. et al | 2020 | Spain | Prospective cohort | Fulfillment of the diagnostic criteria of FFA | 24/57 | / | / | All women | 62.4 | Prospectively recruited patients | / | 7 |
| Pindado-Ortega, C. et al | 2018 | Spain | Cross-sectional | Clinically and using trichoscopy, with histologic confifirmation in atypical cases | 35/103(ETR:28; PPR:7) | / | / | All women | 55.8 | Clinic patients | / | 9 |
| Maldonado Cid, P. et al | 2020 | Spain | Retrospective  cross-sectional | Clinically and using trichoscopy, with histologic confifirmation in atypical cases | 15/75 | / | / | 2: 73 | 61 | Clinic patients | / | 9 |
| Kanti, V. et al | 2019 | Germany | Cross-sectional | By dermatologist, clinically and using trichoscopy, with histologic confifirmation in atypical cases | 9/490(M: 3/23; F: 6/467) | / | / | 23: 467 | 65 | Clinic patients | / | 8 |
| Dorgham, N. A. et al | 2022 | Egypt | Retrospective trial | By medical records | 2/58 | / | / | All women | 50.48 | Clinic patients | / | 8 |
| Doche, I. et al | 2021 | Brazil | Multicenter retrospective study | By medical records | 11/33 | / | / | All men | 53.1 | Clinic patients | / | 8 |

FFA: frontal fibrosing alopecia; OR: odds ratio; CI: confidence intervals; M: male; F: female; ETR: erythematous rosacea; PPR: papulopustular rosacea
