# Supplementary material for: Association between frontal fibrosing Alopecia and Rosacea: Results from clinical observational studies and gene expression profiles
Source: Front Immunol. 2022 Aug 24;13:985081. doi: 10.3389/fimmu.2022.985081 (PMC9448884; doi:10.3389/fimmu.2022.985081)
Supplement: Supplementary file 3 [file Table_2.docx]

**Supplementary Table 2. Quantitative assessment results of Begg’s test**

|  | The odds of rosacea in patients with FFA | The prevalence of rosacea in patients with FFA | The prevalence of rosacea in male patients with FFA | The prevalence of rosacea in female patients with FFA |
| --- | --- | --- | --- | --- |
| P- value | 0.296 | 0.251 | 1.000 | 0.133 |
