# Supplementary material for: Association between frontal fibrosing Alopecia and Rosacea: Results from clinical observational studies and gene expression profiles
Source: Front Immunol. 2022 Aug 24;13:985081. doi: 10.3389/fimmu.2022.985081 (PMC9448884; doi:10.3389/fimmu.2022.985081)
Supplement: Supplementary file 4 [file Table_3.docx]

**Supplementary Table 3. Details of the hub genes**

| Gene | Name | Function |
| --- | --- | --- |
| CCL19 | C-C Motif Chemokine Ligand 19 | May play a role not only in inflammatory and immunological responses but also in normal lymphocyte recirculation and homing. May play an important role in trafficking of T-cells in thymus, and T-cell and B-cell migration to secondary lymphoid organs. Binds to chemokine receptor CCR7. Recombinant CCL19 shows potent chemotactic activity for T-cells and B-cells but not for granulocytes and monocytes. Binds to atypical chemokine receptor ACKR4 and mediates the recruitment of beta-arrestin (ARRB1/2) to ACKR4. |
| CCR5 | C-C Motif Chemokine Receptor 5 | Receptor for a number of inflammatory CC-chemokines including CCL3/MIP-1-alpha, CCL4/MIP-1-beta and RANTES and subsequently transduces a signal by increasing the intracellular calcium ion level. May play a role in the control of granulocytic lineage proliferation or differentiation. Participates in T-lymphocyte migration to the infection site by acting as a chemotactic receptor |
| CD2 | CD2 Molecule | CD2 interacts with lymphocyte function-associated antigen CD58 (LFA-3) and CD48/BCM1 to mediate adhesion between T-cells and other cell types. CD2 is implicated in the triggering of T-cells, the cytoplasmic domain is implicated in the signaling function. |
| CD38 | CD38 Molecule | Synthesizes the second messengers cyclic ADP-ribose and nicotinate-adenine dinucleotide phosphate, the former a second messenger for glucose-induced insulin secretion. Also has cADP hydrolase activity. Also moonlights as a receptor in cells of the immune system. |
| CD83 | CD83 Molecule | May play a significant role in antigen presentation or the cellular interactions that follow lymphocyte activation |
| CXCL10 | C-X-C Motif Chemokine Ligand 10 | Pro-inflammatory cytokine that is involved in a wide variety of processes such as chemotaxis, differentiation, and activation of peripheral immune cells, regulation of cell growth, apoptosis and modulation of angiostatic effects |
| CXCL11 | C-X-C Motif Chemokine Ligand 11 | Chemotactic for interleukin-activated T-cells but not unstimulated T-cells, neutrophils or monocytes. Induces calcium release in activated T-cells. Binds to CXCR3. May play an important role in CNS diseases which involve T-cell recruitment. May play a role in skin immune responses |
| CXCL8 | C-X-C Motif Chemokine Ligand 8 | IL-8 is a chemotactic factor that attracts neutrophils, basophils, and T-cells, but not monocytes. It is also involved in neutrophil activation. It is released from several cell types in response to an inflammatory stimulus. IL-8(6-77) has a 5-10-fold higher activity on neutrophil activation, IL-8(5-77) has increased activity on neutrophil activation and IL-8(7-77) has a higher affinity to receptors CXCR1 and CXCR2 as compared to IL-8(1-77), respectively. |
| CXCL9 | C-X-C Motif Chemokine Ligand 9 | Cytokine that affects the growth, movement, or activation state of cells that participate in immune and inflammatory response. Chemotactic for activated T-cells. Binds to CXCR3. |
| CXCR4 | C-X-C Motif Chemokine Receptor 4 | Binds bacterial lipopolysaccharide (LPS) et mediates LPS-induced inflammatory response, including TNF secretion by monocytes |
| IRF1 | Interferon Regulatory Factor 1 | Transcriptional regulator which displays a remarkable functional diversity in the regulation of cellular responses |
| IRF8 | Interferon Regulatory Factor 8 | Transcription factor that specifically binds to the upstream regulatory region of type I interferon (IFN) and IFN-inducible MHC class I genes |
| PTPRC | Protein Tyrosine Phosphatase Receptor Type C | Protein tyrosine-protein phosphatase required for T-cell activation through the antigen receptor. Acts as a positive regulator of T-cell coactivation upon binding to DPP4. The first PTPase domain has enzymatic activity, while the second one seems to affect the substrate specificity of the first one. Upon T-cell activation, recruits and dephosphorylates SKAP1 and FYN. Dephosphorylates LYN, and thereby modulates LYN activity (By similarity). |
