# Supplementary material for: Association between frontal fibrosing Alopecia and Rosacea: Results from clinical observational studies and gene expression profiles
Source: Front Immunol. 2022 Aug 24;13:985081. doi: 10.3389/fimmu.2022.985081 (PMC9448884; doi:10.3389/fimmu.2022.985081)
Supplement: Supplementary file 5 [file Table_4.docx]

**Supplementary Table 4. Key transcription factors found on the transcriptional regulatory relationships unraveled by sentence-based text-mining database**

| Key TF | Description | of overlapped genes | P value |
| --- | --- | --- | --- |
| RELA | v-rel reticuloendotheliosis viral oncogene homolog A (avian) | CCL19, CCR5, CD38, CD83, CXCL10, CXCL8, CXCR4, IRF1 | 4.57E-12 |
| NFKB1 | nuclear factor of kappa light polypeptide gene enhancer in B-cells 1 | CCL19, CCR5, CD38, CD83, CXCL10, CXCL8, CXCR4, IRF1 | 4.82E-12 |
| STAT1 | signal transducer and activator of transcription 1, 91kDa | CXCL10, IRF1, IRF8 | 0.0000236 |
| KLF2 | Kruppel-like factor 2 (lung) | CCR5, CXCR4 | 2.4E-05 |
| ERG | v-ets erythroblastosis virus E26 oncogene homolog (avian) | CXCL8, CXCR4 | 0.000152 |
| USF2 | upstream transcription factor 2, c-fos interacting | CD2, CXCR4 | 0.000465 |
| USF1 | upstream transcription factor 1 | CD2, CXCR4 | 0.000889 |
| YY1 | YY1 transcription factor | CCR5, CXCR4 | 0.00173 |
| MYC | v-myc myelocytomatosis viral oncogene homolog (avian) | CD38, CXCR4 | 0.00209 |
| STAT3 | signal transducer and activator of transcription 3 (acute-phase response factor) | CXCL8, IRF1 | 0.00415 |
